# Supraphysiological androgen levels induce cellular senescence in human prostate cancer cells through the Src-Akt pathway

**DOI:** 10.1186/1476-4598-13-214

**Published:** 2014-09-12

**Authors:** Julia Roediger, Wiebke Hessenkemper, Sophie Bartsch, Marina Manvelyan, Soeren S Huettner, Thomas Liehr, Mohsen Esmaeili, Susan Foller, Iver Petersen, Marc-Oliver Grimm, Aria Baniahmad

**Affiliations:** Institute of Human Genetics, Jena University Hospital, 07740 Jena, Germany; Institute of Pathology, Jena University Hospital, 07740 Jena, Germany; Institute of Urology, Jena University Hospital, 07740 Jena, Germany; National Institutes of Health (NIH) Section on Molecular Morphogenesis, Bethesda, MD 20892-5431 USA

**Keywords:** Nuclear receptor, Non-genomic signaling, Tumor suppression, Cellular senescence, Autophagy

## Abstract

**Background:**

Prostate cancer (PCa) is the second leading cause of cancer mortality of men in Western countries. The androgen receptor (AR) and AR-agonists (androgens) are required for the development and progression of the normal prostate as well as PCa. However, it is discussed that in addition to their tumor promoting activity, androgens may also exhibit tumor suppressive effects. A biphasic growth response to androgens a growth-promoting and -inhibition has been observed that suggests that administration of supraphysiological androgen levels mediates growth reduction in AR expressing PCa cells.

**Methods:**

Detection of senescence markers, three dimensional interphase fluorescence in situ hybridization (3D-iFISH), qRT-PCR, Western blotting, detection of GFP fusions, prostatectomy, *ex vivo* culturing.

**Results:**

Here, we describe that supraphysiological levels of androgens induce cell cycle arrest and markers of cellular senescence in human PCa cells, which may in part explain the growth inhibitory role of androgens. The expression of the senescence associated beta galactosidase is observed by treatment with the natural androgen DHT or the less metabolized synthetic androgen R1881. The induction of senescence marker was detected in human PCa cell lines as well as in human primary PCa tissue derived from prostatectomy treated *ex vivo*. Using interphase FISH (iFISH) suggests that the androgen-induced cellular senescence is associated with localizing the genomic E2F1 locus to senescence associated heterochromatic foci. Analysis of different signaling pathways in LNCaP cells suggest that the p16-Rb-E2F1 pathway is essential for the induction of cellular senescence since treatment with siRNA directed against p16 reduces the level of androgen-induced cellular senescence. Based on the rapid induction of androgen-mediated cellular senescence we identified the Src-PI3K-Akt-signaling pathway and autophagy being in part involved in androgen regulation.

**Conclusions:**

Taken together, our data suggest that AR-agonists at supraphysiological levels mediate induction of cellular senescence in human PCa cells, which may have a protective anti-cancer role. These results provide also new insights for understanding androgen-mediated regulation of PCa growth.

**Electronic supplementary material:**

The online version of this article (doi:10.1186/1476-4598-13-214) contains supplementary material, which is available to authorized users.

## Background

Prostate Cancer (PCa) is an important age-related diseases being the most common cancer malignancy and the second leading cause of cancer mortality in men in western countries [1]. Initially, PCa progression is androgen receptor (AR)- and androgen-dependent. Unfortunately, after 12–18 months of hormone ablation therapy the advanced PCa growth is becoming androgen-independent but remains dependent on AR [2], which indicates the importance of developing new therapeutic strategies. Interestingly, it is known that with increased age the androgen level is decreasing, which seems to be timely associated with increased risk of PCa [3, 4]. It has been suggested that androgens first have a protective role for prostate proliferation [4–8]. In line with this, Niu *et al.* [9] revealed using a mouse model that the functional AR exhibits both proliferation promoting as well as tumor suppressive functions. However androgen-mediated growth inhibition is less examined and not well understood, and thus it is postulated that androgen administration could reduce PCa growth.

Cellular senescence is an irreversible cell cycle arrest mediated through exogenous and endogenous stimuli, which cause changes in cell morphology and gene expression profiles [10, 11]. New insights reveal that cellular senescence occurs during embryogenesis as a normal programmed mechanism that plays instructive roles in development and controls patterning [12, 13]. It is suggested that this cellular program may be reactivated during early premalignant carcinogenesis as a protective cellular mechanism to prevent malignant cancer. Therefore, the proliferation arrest of senescent cells has been indicated to act tumor suppressive. In malignant cells, however, this program of cell cycle arrest by cellular senescence seems to be inhibited [14]. Hence, the process of cellular senescence represents a natural defense mechanism against tumor progression and thus the exogenous re-activation and induction of cellular senescence is a potential target for cancer therapy [15].

Tumor suppressor proteins and their signaling pathways such as the p14-p53-p21 and p16-pRb-E2F1 pathways are involved in the induction of cellular senescence [16–19]. Further, autophagy, a highly conserved, lysosome-mediated process that degrades cytoplasmic components, seems to be linked to the initiating of cellular senescence [20, 21]. Cellular senescence is also associated with changes in the nuclear chromatin structure to generate senescence-associated heterochromatic foci (SAHF) as another marker for cellular senescence [22].

The AR belongs to the nuclear hormone receptor superfamily. Besides its function as a ligand-controlled transcription factor the AR is also to induce ligand-mediated so called rapid signaling in the cytoplasm such as the MAP-kinase and the Src tyrosine kinase signaling [23–27].

Here, our data suggest that androgens induce cellular senescence in a concentration-dependent manner in human PCa cell lines, which may explain the growth inhibitory role of androgens. This is confirmed by *ex vivo* studies with primary human PCa biopsy material, where androgens induce cellular senescence in malignant human PCa tissue. Furthermore, we observed that besides the tumor suppressors p16, pRb also Src - Akt, mediate the androgen-mediated induction of cellular senescence. The data provide molecular insights into androgen-mediated cellular senescence representing important principles to understand the role of AR-signaling as a target of PCa therapy.

## Results & discussion

### AR-agonists induce cellular senescence in a concentration-dependent manner in PCa cell lines

AR-agonists are known to promote prostate development as well as PCa growth [28]. However, Sonnenschein *et al*. [29] described a concentration-dependent proliferation arrest in PCa cells after treatment with the natural agonist DHT or the synthetic R1881 at supraphysiological levels. Notably, the underlying cellular and molecular mechanisms are still unclear. Therefore, we hypothesized that androgens may induce a pathway of cellular senescence.

**Figure 1:**
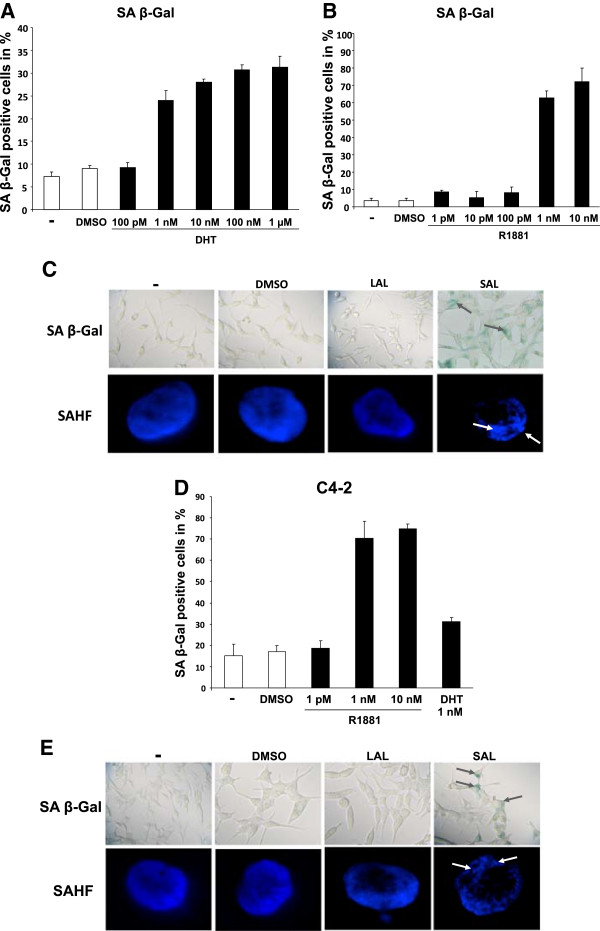
**AR-agonists induce cellular senescence in LNCaP and C4-2 human prostate cancer cell lines in a concentration dependent manner.** Androgen dependent growing LNCaP and castration resistant C4-2 PCa cell lines were incubated with DMSO as solvent control and different concentrations of the synthetic and more stable androgen R1881 or the natural androgen DHT for 72 h. Because DHT is metabolized rapidly, DHT was added daily. 1 pM R1881 is defined as low androgen levels (LAL) and 1 nM R1881 as supraphysiological androgen levels (SAL). Cells were fixed and analyzed for SA β-Gal activity using a light microscopy and 3x 200 cells were counted and as means of the triplets in percent diagramed. **A**. SA β-gal activity of LNCaP cells treated with R1881 (methyltrienolone). **B**. SA β-gal activity of LNCaP cells treated with DHT. **C**. SA β-Gal staining in LNCaP cells at 200x magnification by phase microscopy. Upper panel: Arrowheads indicate the blue precipitations in the cytoplasm in senescent cells. Lower panel depicts the formation of senescence associated heterochromatic foci (SAHF) via DAPI staining of the nucleus. For fluorescence microscopy a 1000x magnification was used. Arrowheads mark accumulation of heterochromatic foci. **D**. SA β-Gal activity of the human castration resistant PCa cells C4-2. **E**. Microscopy of cells after SA β-Gal activity detection (upper panel) and DAPI staining with SAHF formation (lower panel) after androgen treatment of C4-2 cells.

Androgen-dependent growing LNCaP cells were treated with DHT and R1881 for 3 days and through the measurement of SA β-Gal activity the induction of cellular senescence was analyzed. Interestingly, we observed that both the natural and the synthetic androgen induce cellular senescence in a concentration-dependent manner. Administration of 1 nM R1881 or 1 nM DHT indicate a strong induction of SA β-Gal activity, in contrast, lower androgen levels show the basal level of cellular senescence similar to the untreated or the solvent control (Figure 1A, B). Higher concentrations of R1881 result in a higher percentage of cells expressing this marker compared to the natural compound DHT indicating a higher potency to induce cellular senescence. An explanation for this might be that in addition to a higher affinity for the AR, R1881 as a synthetic androgen is not metabolized as rapidly as the natural DHT [30]. Therefore, R1881 was used for further studies. Based on these results we defined here 1nM R1881 as supraphysiological androgen level (SAL) and 1pM R1881 concentration as low androgen level (LAL). Longer treatment periods did not increase the level of SA β-Gal activity indicating that 3 days of treatment with SAL is sufficient to induce cellular senescence (Additional file 1: Figure S1).

To confirm the androgen-induced cellular senescence, we examined a further marker, the formation of senescence-associated heterochromatic foci (SAHF). DAPI staining of the treated cells revealed that SAL treatment induces an accumulation of heterochromatin in LNCaP cells (Figure 1C). Similar results were obtained in androgen-independent growing C4-2 cells where androgen treatment also induced both the SA β-Gal activity and the formation of SAHFs (Figure 1D, E). Moreover, using PC3-AR cells expressing the human AR we observed induction of cellular senescence under SAL but not LAL conditions (Additional file 2: Figure S2A), whereas we did not observe an androgen-induced cellular senescence using non-AR expressing PC3 cells (Additional file 2: Figure S2B). This confirms that androgen-induced cellular senescence is AR-dependent PCa cell lines.

**Figure 2:**
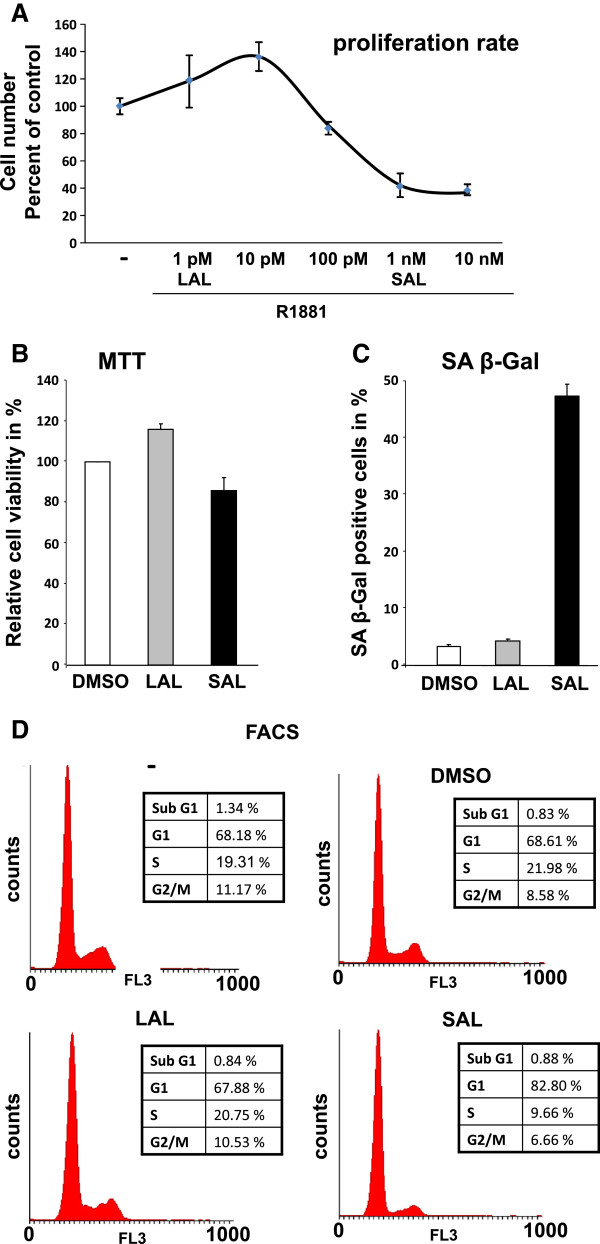
**Higher androgen levels induce growth inhibition and G1 arrest in LNCaP cells.** LNCaP cells were treated for 72 h with 1 pM R1881 defined as low androgen levels (LAL) and 1 nM R1881 as supraphysiological androgen levels (SAL). **A**. SAL treatment inhibits growth of LNCaP cells. Cells were treated with the indicted concentrations of R1881 for three days. Cell number was determined and plotted against the untreated control. For each time point n = 4, the errors are shown in SEM. **B**. Viability analysis of treated cells was measured by the MTT assay. LNCaP cells were treated for 72 h with DMSO or with the indicated concentrations of R1881 following spectrometrical measurement to analyze the relative cell viability. Data represent the mean from triplets and show the cell viability in percent. **C**. To analyze the irreversibility of androgen-induced cellular senescence LNCaP cells were treated with SAL or LAL as well as DMSO for 72 h. Afterwards compounds were removed and cells were cultured for additional 72 h, followed by fixation and determination of the SA β-gal activity level via light microscopy at a 200x magnification. 3x 200 cells from triplets were counted and their means in percent diagramed. **D**. FACS analysis was performed to analyze the cell cycle state of R1881 treated cells. LNCaP cells were incubated for 72 h with solvent control or R1881 (SAL or LAL) and stained with propidium iodide followed by cell sorting analysis. The acquired FACS data were analyzed by Cylchred (Ormerod, Hoy). Counts of the different cell cycle phases were represented in percent.

Growth analyses were performed to analyze the relation between the proliferation rate of LNCaP cells and the concentration of the androgen R1881 (Figure 2A). The data indicate that at LAL the cell number of the androgen-dependent cells increases whereas cells grow less in the presence of SAL. This suggests a growth inhibition at higher, supraphysiological levels of androgens. The observed biphasic growth response upon androgen treatment is in line with previous observations [29, 31] and is in accordance with the observed induction of cellular senescence at supraphysiological androgen levels.To reveal whether SAL changes the cell viability, MTT-assays were performed. The data indicate that treated LNCaP cells remain viable at similar levels as compared to untreated LNCaP cells (Figure 2B). Next, we analyzed whether the activity of the senescent marker that appears after three days is reversible. For this purpose androgens were removed after 3 days by washing the cells and fresh medium without androgens was added for further 3 days. The level of SA β-Gal activity remains unchanged after removal of SAL treatment suggesting that the androgen-induced cellular senescence is irreversible (Figure 2C).In general, cellular senescence is associated with an arrest at the G1/G0 phase of the cell cycle. FACS analyses were performed which indicates that androgen treatment at SAL increases the number of cells in the G1/G0 phase of the cell cycle (Figure 2D), which is in agreement with our data.

Thus, these data suggest that supraphysiological levels of androgens induce markers of cellular senescence and inhibit cell growth in a concentration-dependent manner in both androgen-dependent and -independent growing PCa-cell lines.

### AR-agonists induce cellular senescence in human PCa tissue *ex vivo*

**Figure 3:**
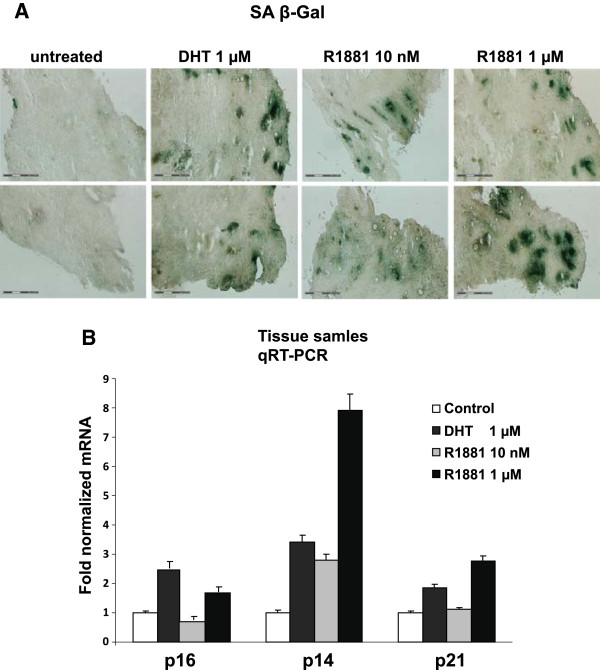
**AR-agonists induce cellular senescence in human PCa tissue** ***ex vivo*** **.** Human prostate cancer tissues after prostatectomy were used to examine the effect of androgens *ex vivo*. PCa tissue (n = 5 patients) and control (n = 2) samples were treated daily with 1 μM DHT or 10 nM and 1 μM R1881 for 48 h. **A**. Pictures of representative SA β-Gal stained cryosections (10 μm thickness) of untreated or treated PCa tumor tissue and non-tumor tissue. Bars represent 300 μm and spots of SA β-gal positive cells were labeled with arrows. **B**. Analysis of the gene expression of p16, p14 and p21 was performed via qRT-PCR. Gene expression was normalized to GAPDH and the values for untreated samples were set as one. Error bars indicate the standard deviation of the mean of doublets of the samples of one group.

To detect whether androgens induce cellular senescence in primary tissue samples, human PCa specimens derived from prostatectomies were treated for 2 days with 10 nM and 1 μM R1881 or 1 μM DHT *ex vivo*. DHT levels in men range between 0.8 – 2.5 nM [32]. However the level decrease by age. R1881 has been shown to be more potent compared to DHT, therefore higher concentrations of DHT or R1881 were used to ensure supraphysiological levels and that the compounds reach the cells within the tissue blocks that had a thickness of about 5 × 5 mm. Interestingly, the SA β-Gal activity was highly increased after androgen administration; in contrast there were only few SA β-Gal positive stained cells detectable in PCa tissue without hormone treatment of the same biopsy (Figure 3A). Differences between the androgens DHT and R1881 were not observed. Thus, the data suggest that androgen treatment leads to the induction of SA β-Gal activity in human PCa tissue *ex vivo*.

Furthermore, RNA was extracted from these tissue samples to analyze the expression level of the tumor suppressor p16, p14 and p21, which are described to be involved in cellular senescence [17–19]. The p14 gene expression was increased after DHT as well as R1881 administration (Figure 3B). p21 and p16 gene expression exhibited an up-regulation by androgens at SAL.

In summary, to our knowledge this is the first time that reveal that androgen treatment is able to induce cellular senescence in human PCa tissue *ex vivo*, which is in line with the hypothesis that PCa may undergo cellular senescence and that the LNCaP cell line can serve well as a suitable *in vitro* senescence model system that represents similarities to *ex vivo* studies using primary human cancer tissue.

### Androgen-induced cellular senescence is mediated through tumor suppressor genes in LNCaP cells

**Figure 4:**
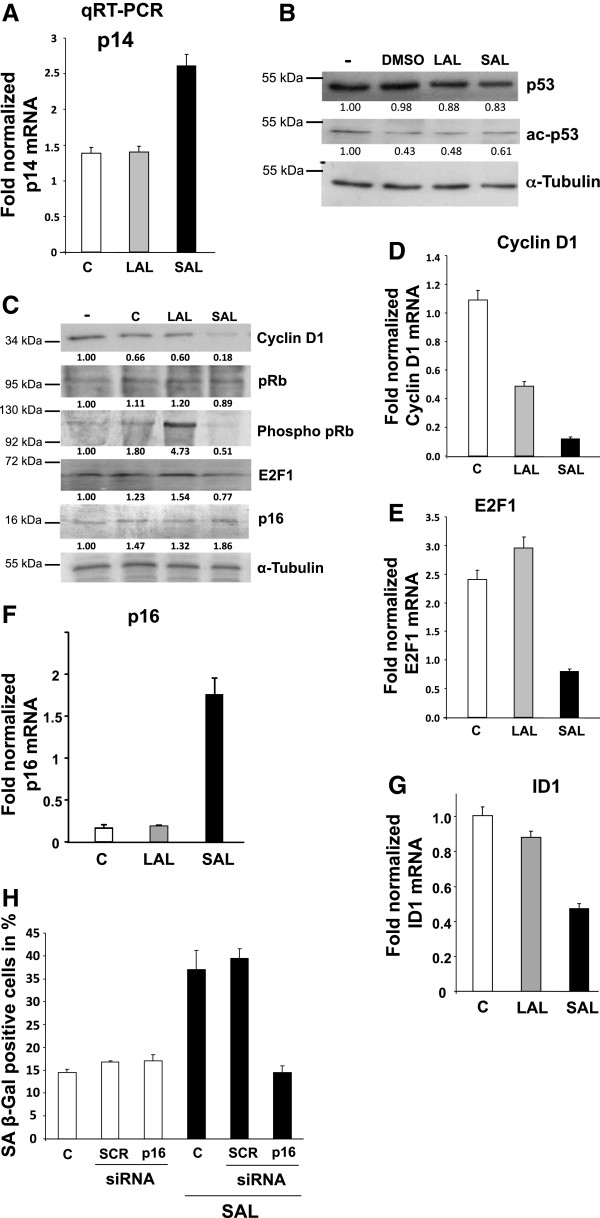
**Androgen-induced cellular senescence is mediated through the tumor suppressors p16-pRb.** To examine the signaling pathways involved in the induction of cellular senescence, Western blotting, 3D-FISH of interphase nuclei, qRT-PCRs and transient transfections with siRNA were performed. LNCaP cells were incubated for 72 h with solvent control or different R1881 concentrations (1 pM = LAL; 1 nM = SAL). C, solvent control (DMSO). **A**. qRT-PCR was performed to analyze mRNA expression of p14. Gene expression was normalized to β-actin and the values for untreated samples (C). Error bars indicate the standard deviation of the mean of doublets. **B**. Protein levels of p53 as a target of p14 were detected with immunoblotting using antibodies against p53 and acetylated p53 (ac p53) known to be involved in cellular senescence pathway. Quantification of the bands was realized via Labimage D1 and the expression levels of the target proteins were normalized and given as band intensity to the loading control α-tubulin, untreated sample. **C**. Changes of the p16-pRb pathway by androgen treatment were analyzed by Western blotting. **D-G)** Quantitative qRT-PCR was performed to analyze mRNA expression with primers directed against indicated mRNAs. Gene expression was normalized to β-actin. Error bars indicate the standard deviation of the mean of doublets. C: solvent control (DMSO). **H**. Transient transfection of small interference RNA (siRNA) and subsequent SA β-gal assays were used to analyze the effects of p16 on the androgen-induced senescence. LNCaP cells were transfected via electroporation with srambled control (SCR) siRNA as negative control or p16 siRNA and without any siRNA (-) 24 h prior R1881 treatment. Thereafter, cells were fixed and analyzed for SA β-Gal activity using a light microscopy at 200x magnification and 3× 200 cells were counted and as means of the triplets in percent diagramed.

The p14 gene expression, an activator of p53 via the inhibition of Mdm2, was up-regulated in the PCa tissue *ex viv*o upon androgen treatment. To examine the role of this pathway we analyzed mRNA expression after administration of androgens in LNCaP cells. The gene expression of p14 is also increased at SAL but not at LAL (Figure 4A). An acetylation and stabilization of the tumor suppressor p53 has been described to occur by senescence-inducing stimuli [33]. However, neither the total nor acetylated protein levels of p53 seem to be changed after androgen treatment in comparison to DMSO as solvent control (Figure 4B), indicating that p53 might not be involved in the androgen-mediated cellular senescence.

p16, as a cyclin-dependent kinase inhibitor, is known to mediate a hypophosphorylation of pRb and consequently a down regulation of the E2F1 transactivation as wells as the E2F1 gene expression [33]. After administration of SAL an upregulation of p16, hypophosphorylation of pRb and down-regulation of the pRb targets Cyclin D1 as well as of E2F1 protein levels were observed indicating that the p16-pRb pathway is regulated by SAL treatment (Figure 4C). Similar results were obtained by treating the cells for 6 days (Additional file 3: Figure S3). In contrast, LAL treatment mediated no detectable changes of p16, Cyclin D1 and E2F1 expression level. In line with this, SAL treatment led to inhibition of down-stream targets of pRB, Cyclin D1 as well as E2F1 at mRNA level (Figure 4D, E), whereas the p16 mRNA is upregulated by SAL doses (Figure 4F). Accordingly, the mRNA level of ID1, an inhibitor of p16 expression, is reduced upon SAL administration (Figure 4G). Thus, these data indicate that the p16-pRb-E2F1 pathway is associated with the androgen-mediated cellular senescence.Interestingly, transient knock-down of p16 by siRNA strongly reduces the R1881-mediated level of SA β-Gal activity compared to scrambled (SCR) siRNA, whereas knock-down of p16 without androgen treatment results in basal level similar to the controls (Figure 4H) indicating that the androgen-induced cellular senescence is in part mediated by the induction of p16.

**Figure 5:**
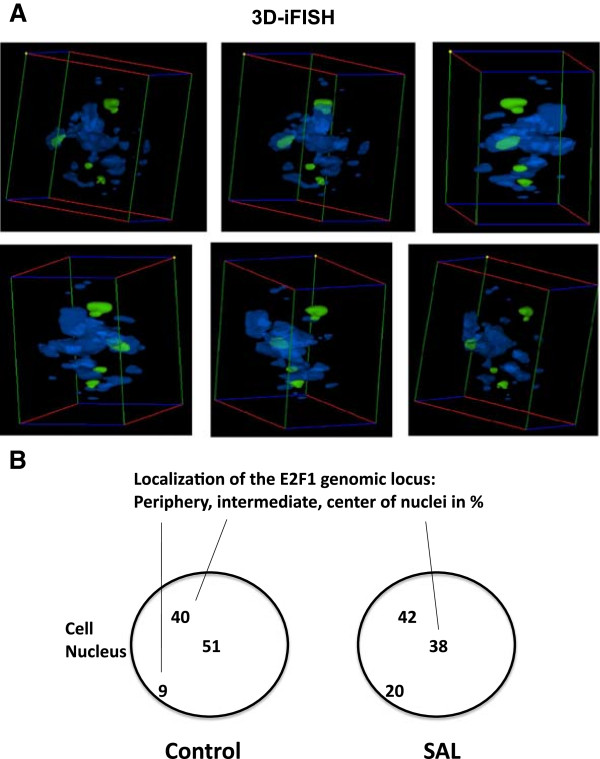
**Three dimensional interphase FISH (3D-iFISH) indicates an androgen-induced change of the nuclear localization of the E2F1 loci under SAL conditions. A**. 3D-iFISH of interphase LNCaP nuclei using a DNA probe directed against the genomic E2F1 gene loci (chromosome 20) were performed and costained with DAPI to detect the location of the genomic locus in relation to formed SAHFs after SAL treatment. One nucleus is shown representatively at various angles for 3D projection. Analysis of 24 nuclei suggests that 65% of the E2F1 loci colocalize with SAHFs (see text). **B**. Analysis of 24 senescent LNCaP nuclei in the same experimental setup as in (I) summarizing the location of the E2F1 loci within the nuclei differentiating between a peripheral, intermediate and center localization of the E2F1 genomic loci on chromosome 20.

It has been suggested that the formation of SAHFs coincides with stable repression of E2F target genes in a pRb-dependent manner [34]. SAHFs are considered as heterochromatin, which is also found perinuclear. Since E2F1 regulates the expression of its own gene by a positive feedback loop, we analyzed whether the human E2F1 gene loci localize to SAHF vicinity and whether the E2F1 locus changes its position within the cell nucleus. For that purpose we used interphase 3D-FISH (3D-iFISH) to label the E2F1 locus on chromosome 20 and counterstained with DAPI to detect SAHFs of interphase LNCaP cells. SAHFs were sparely detected in control treatment. SAL treatment indicated that the FISH signals are in the vicinity of SAHFs (Figure 5A). Analyzing 24 interphase 3D nuclei we found that 65% of the E2F1 covering FISH-signals are colocalizing with SAHFs and 35% of the FISH signals lie outside of SAHFs (data not shown, Figure 5B), which indicates an enrichment of the genomic locus within the SAHFs. Interestingly, analyzing the location of the E2F1 loci in the control treated group reveals a preferred central and intermediate localization of the E2F1 loci, whereas SAL treatment indicates an increase of FISH signals in the nuclear periphery (Figure 5B), suggesting an androgen-induced change of the nuclear localization of the E2F1 loci under SAL conditions.

Taken together, the data strongly indicate that androgen treatment at SAL induces cellular senescence through the induction of the p16-pRB-E2F1 pathway.

### Rapid signaling participates in androgen-induced cellular senescence

**Figure 6:**
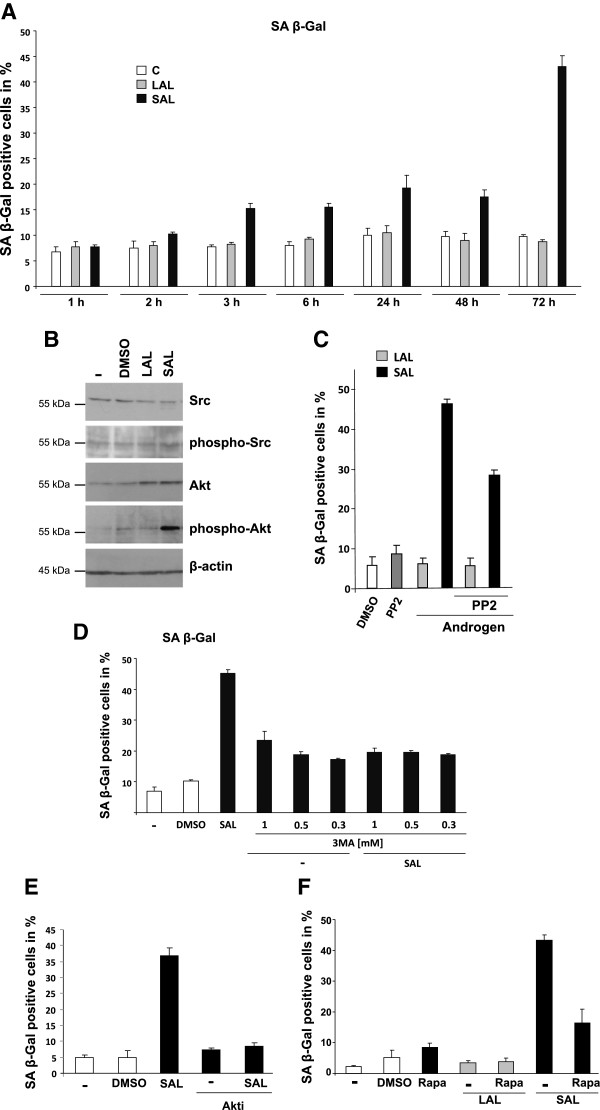
**Supraphysiological androgen levels mediate cellular senescence in LNCaP cells partially through rapid signaling.** The induction of cellular senescence in LNCaP cells induced by androgens was determined after indicated treatment times with solvent control, 1 pM (low androgen levels = LAL) or 1 nM R1881 (supraphysiological androgen level = SAL). After the treatment times cells were washed to remove the compounds and cultured for total of 3 days. Afterwards, cells were fixed and analyzed for SA β-Gal activity using a light microscopy at 200x magnification and 3x 200 cells were counted and as means of the triplets plotted in percent. **A**. LNCaP cells were treated between 1 for up to 72 h. Thereby at shorter incubation times, indicated compounds were removed and cells were cultivated until 72 h in total. **B**. LNCaP cells were incubated for 72 h with R1881 and followed by detection of Akt and Src as well as their phosphorylated forms by specific antibodies by Western blot analysis. For quantification LabImage D1 was used. **C**. Detection of SA β-gal staining of LNCaP cells incubated for 72 h with the Src tyrosine kinase inhibitor PP2 (1 μM) in combination with LAL and SAL. **D**. The PI3-kinase inhibitor 3-MA was added to LNCaP cell culture at the indicated concentrations with and without R1881 for 3 d and analyzed using light microscopy and 3x 200 cells were counted and the mean of the triplets is diagrammed in percent. **E**. Detection of SA β-Gal staining of LNCaP cells incubated for 72 h with an Akt-inhibitor (Akti; 1 μM) in combination with SAL treatment. **F**. Detection of SA β-Gal staining of LNCaP cells incubated for 72 h with the mTOR inhibitor rapamycin (1 nM) in combination with LAL or SAL treatment.

We sought to analyze shorter incubation times of androgens to investigate the minimal treatment time for androgen-induced cellular senescence. Administration of androgens at LAL reveals no changes in the level of cellular senescence at any time point. In contrast, the SA β-Gal activity under SAL conditions is increased after only 3 h of treatment and reaches a maximum after 72 h (Figure 6A). These data indicate that the R1881-induced cellular senescence is partially mediated through a rapid signaling response.On the one hand, Western blotting data indicate that the total amount of Src seems to be unchanged after SAL treatment (Figure 6B). The ratio between Src and phospho-Src is changed towards a slight increase of Src phosphorylation after SAL. Interestingly, we also observed an increase of Akt phosphorylation (Figure 6B). Based on these findings we investigated the involvement of the Src- and Akt-kinase in androgen-induced cellular senescence using first a Src-specific inhibitor. Notably, treatment of LNCaP cells with the Src inhibitor PP2 under SAL conditions reduces the androgen-mediated cellular senescence (Figure 6C). In contrast, inhibition of Src without androgens or with LAL has no detectable influence on the SA β-Gal activity in LNCaP cells compared to control. This indicates that the androgen-induced cellular senescence is mediated in part by Src-signaling.

On the other hand, blocking MEK1/2-kinases, which function downstream of the Src-kinase, via the U0126 inhibitor reveals no obvious effect on androgen-induced cellular senescence. In line with this, R1881 mediates no change of the phosphorylation level of ERK1/2 (Additional file 4: Figure S4). Furthermore, other Src downstream factors such as p38 and STAT3 were analyzed using specific inhibitors without an indication for their participation in this process (data not shown). With this background we focused on other pathways downstream of the Src-kinase.Akt phosphorylation is a well-known pathway of the Src tyrosine kinase and involves signaling molecules such as the PI3K as well as the mammalian target of rapamycin (mTOR). Using inhibitors the role of these factors in androgen-mediated cellular senescence was analyzed. Inhibition of PI3K, an Akt-activating kinase, by the 3-MA inhibitor, reduced the level of androgen-induced cellular senescent cells (Figure 6D), which confirms that the Src-Akt signaling pathway is involved in androgen-induced cellular senescence at supraphysiological levels. Similarly, using a specific Akt-kinase inhibitor (Akti) reveals a strong reduction of the SAL-mediated SA β-Gal activity (Figure 6E). Thereby, treatment with the Akt-inhibitor alone results in a basal level of cellular senescence similar to untreated or DMSO control, whereas in combination with SAL the androgen-mediated cellular senescence is strongly reduced and close to the basal level. Since the incubation at SAL but not at LAL specifically increases the phosphorylation level of Akt protein as well as inhibition of this pathway reduces androgen-induced cellular senescence, we assume that the Akt-kinase could be one key regulator of androgen-induced cellular senescence in LNCaP cells.

Thus, the data suggest that the Src tyrosine kinase and the downstream Akt-PI3K pathway mediate in part the androgen-induced cellular senescence.

A further downstream target of the Src- and the Akt-kinase is mTOR, which is involved in proliferation and cell cycle regulation processes [35]. Rapamycin alone mediates no detectable change in the level of cellular senescence. In contrast, rapamycin co-treated with SAL resulted in reduction of SA β-Gal positive stained cells (Figure 6F).

Notably, AR translocaton studies using a GFP-AR expression plasmid indicate that neither the Src-kinase inhibitor PP2 nor the mTOR inhibitor rapamycin seem to have an influence on the receptor nuclear translocation (data not shown). Thus, the data show that rapamycin reduces the androgen-mediated SA β-Gal activity and suggest that mTOR is partially involved in androgen-mediated cellular senescence. This supports the notion that the Src-Akt-mTOR signaling mediates the androgen-mediated induction of cellular senescence.

Taken together, these observations indicate that androgen-induced cellular senescence is mediated at least in part by the AR-driven rapid signaling pathway involving the Src-Akt-mTOR signaling.

### Androgens regulate autophagy acivity in LNCaP cells

Rapamycin was described to induce the degradation process autophagy [36]. Gamerdinger *et al.* [20] linked this process to cellular senescence. Since rapamycin is reducing SAL-mediated cellular senescence in LNCaP cells, we therefore hypothesized a link between androgen-induced cellular senescence and autophagy.

**Figure 7:**
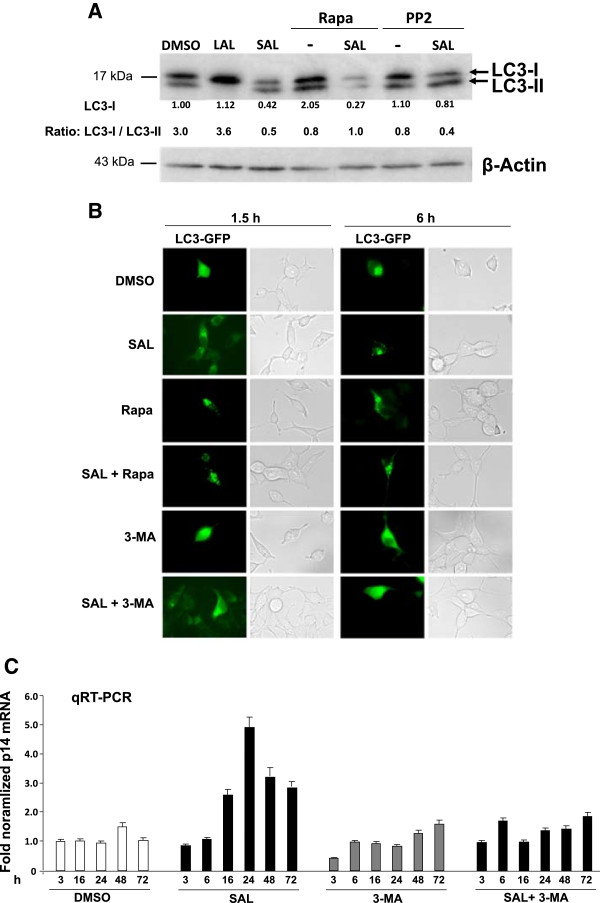
**Androgen-mediated cellular senescence is linked to autophagy activity.** To examine the association between the androgen-mediated induction of SA β-gal activity as a specific marker for cellular senescence and autophagy the conversion of LC3 was analyzed after 3 d incubation of LNCaP cells with SAL or LAL. **A**. The conversion of LC3 I to LC3 II as a marker of autophagy activity was detected by Western blotting experiments. LNCaP cells were treated with the Src tyrosine kinase inhibitor PP2 (1 μM) or the mTOR inhibitor rapamycin (1 nM) in addition to SAL and the conversion of the autophagy marker from LC3 I to LC3 II was detected via Western blotting. Quantification of the bands was performed by the Labimage D1 program. The expression levels of LC3 I and II were normalized to the band intensities of the loading untreated sample control β-actin that was set as 1. The ratios of these values are indicated below. **B**. The puncated pattern of LC3 is indicative for autophagy activity and was analyzed by using GFP-LC3. LNCaP cells were transfected with GFP-LC3 and treated with the indicated compounds for the indicated period. The distribution of GFP-LC3 and formation of puncated pattern was analyzed by fluorescence microscopy. **C**. The up-regulation of p14 gene expression by SAL is inhibited by an inhibitor of PI3K and autophagy. qRT-PCR of LNCaP cells that were treated with the indicated time periods with and without SAL and 3-MA as described in Figure 3B.

The conversion of LC3 was analyzed, which is an important marker of autophagy activity, derived from the cytosolic LC3-I into the autophageosom-associated LC3-II [37]. Accordingly, autophagy activity is measured by the ratio of LC3-I to LC3-II. LC3-II is detectable in the control (Figure 7A) suggesting basal autophagy activity in LNCaP cells. After LAL incubation LC3 conversion is reduced and the protein level of LC3-I is increased (Figure 7A). The different levels of LC3-I may derive from reduced conversion/degradation or protein stability/expression. Interestingly, the data also reveal a promotion of the conversion of LC3-I into LC3-II after SAL administration, indicated by the higher level of LC3-II compared to LC3-I (Figure 7A). These results lead to the assumption that supraphysiological levels of androgens influence autophagy markers and autophagy activity. The strong reduction of the ratio is in part reverted by treatment with rapamycin or the Src inhibitor PP2, which is associated with the inhibition of SAL-mediated SA β-Gal activity. This indicates that autophagy is associated with the SAL-mediated cellular senescence.

Furthermore, a punctuated LC3 pattern, which is indicative of the integration of LC3 as protein aggregates into the autophageosome membrane and therefore an autophagy marker [38] was employed using the GFP-LC3 fusion protein. Interestingly, SAL treatment induced a specific GFP pattern, which is still visible in combination with the autophagy inducer rapamycin but was inhibited through 3-MA co-treatment (Figure 7B), indicating that SAL treatment is associated with enhanced autophagy. Thus inhibition of PI3K and autophagy by 3-MA reduces the level of androgen-mediated cellular senescence.Since androgens induce a robust induction of p14 gene expression in LNCaP cells (Figure 3B) as an androgen response, we analyzed the androgen-induced expression of p14 after shorter treatment times. In the presence or absence of 3-MA time-dependent experiments suggest that under SAL conditions the induction of p14 mRNA expression is observed after only 16 hours (Figure 7C) indicating that p14 may not be the timely primary target of the rapid response to androgen-induced cellular senescence. Interestingly, 3-MA treatment counteracts the androgen-induced p14 gene expression (Figure 7C). Thus, these results suggest that androgens upregulates of autophagy activity, which supports the hypothesis that supraphysiological levels of androgens regulate autophagy activity in LNCaP cells.

Taken together, reduction of SAL-mediated cellular senescence by 3-MA is associated with an inhibition of androgen-induced autophagy activity linking androgen-mediated cellular senescence with autophagy.

## Conclusions

Notably, several reports show that supraphysiological levels of androgens inhibit the proliferation of human PCa cells and tumor growth [5–9]. It is suggested that PCa tumor cells respond in a biphasic manner to androgens. Both at very low and at supraphysiological androgen levels the growth of PCa tumor cells is inhibited. A study on a large prospective cohort, contributes to the gathering evidence that the long standing "androgen hypothesis" of increasing PCa risk with increasing androgen levels might be rejected [39–41]. Furthermore, it has been suggested that the androgen-activated AR acts also as a tumor suppressor for prostate cancer [9]. In line with this, treatment of mice with SAL inhibits the growth of human CRPCa cells in xenograft mouse model system *in vivo* [39, 42].

The androgen-dependent human LNCaP PCa cells, widely used as a PCa model system, display a biphasic proliferative response to androgen stimulation low androgen concentrations stimulate proliferation, while higher androgen concentrations inhibit cell proliferation [43, 44]. However, the underlying molecular mechanism is not fully understood. Here, we suggest that the induction of cellular senescence by supraphysiological androgens may in part explain the inhibition of PCa growth.

Thus, we demonstrate that the induction of cellular senescence markers is detectable in LNCaP cells, the human CRPCa C4-2 cells as well as in human PCa tissue derived from prostatectomies treated with supraphysiological levels of androgens. The induction of the tumor suppressor p16 was observed and accordingly a hypophosphorylation of pRB. E2F1 expression is in part auto-regulated by E2F1 binding sites in the E2F1 promoter [45]. Hypophosphorylated pRb will thus inhibit E2F1-mediated transcriptional activity and thus E2F1 mRNA expression, which is reflected by the observed data. Since siRNA of p16 reduces the level of senescent cells we therefore hypothesize that supraphysiological androgens mediate cellular senescence in part through the p16-pRB-E2F1 pathway. In line with this, we observed an accumulation of the E2F1 gene locus in SAHFs.

Based on the surprisingly rapid response of androgen exposure to induce cellular senescence and the use of inhibitors of the tyrosine kinase family, Akt and mTOR, that inhibit the androgen-induced cellular senescence, we furthermore suggest that the rapid, non-genomic androgen action is involved in the induction of cellular senescence by supraphysiological androgens.

The induction of cellular senescence in human PCa cells has been described by our group using novel AR-specific antagonists addressing the human AR [46] being the first report an AR-dependent induction of cellular senescence. The induction of cellular senescence has been described for PC3 cells, a human metastatic PCa cell line that have originally lost AR expression, stably transfected with the human AR and treated with the androgen R1881, defined there as DHT [47]. We confirmed also the androgen-induced cellular senescence in PC3-AR cells. Interestingly PC3-AR cells are p53 negative and the p16 locus in hypermethylated [47, 48] In line with this and with previous report [47] were unable to detect neither p16 nor a regulation of E2F1 levels by SAL or LAL (Additional file 5: Figure S5), whereas we observed an induction of p21 mRNA level by SAL. The lack of p16 expression in PC3-AR cells suggests that the androgen-induced cellular senescence is mediated by a different pathway in these cells and suggests that the AR might have interestingly various cellular pathways to induce androgen-mediated cellular senescence.

Taken together, our data suggest that supraphysiological androgens induce cellular senescence in human PCa tissues as well as the PCa model cell lines LNCaP and C4-2. To our knowledge this is the first description that androgens induce cellular senescence *ex vivo*. Induction of the tumor suppressor p16 and its pathway is one underlying molecular mechanism to induce cellular senescence together with androgen-mediated rapid signaling is involved in mediating androgen-induced cellular senescence.

Since inhibition of PCa growth is a primary goal in therapy, the induction of cellular senescence and cell cycle inhibition represents an interesting option and may explain previous finding that androgens seem to have some beneficial roles.

## Methods

### Cell lines and culture

As a model of human androgen-dependent growing PCa a LNCaP (lymph node prostate cancer) cell line was used. Cells were cultured in RPMI (1640) with 10% FBS, 1% penicillin/streptomycin, 1% sodium pyruvate and 25 mM HEPES (pH 7.8). Additionally, the C4-2 cell line was used to represent androgen-independent growing PCa cells. Cells were cultured in DMEM supplemented with 20% F12, 10% FCS, 5 μg/ml insulin, 5 μg/ml apotransferin, 0.25 μg/ml biotin, 25 μg/ml adenine and 1% penicillin/streptomycin. All cells were cultured in a 5% CO_2_ - 95% air, humidified atmosphere at 37°C. Growth assays were performed as described earlier [49].

### Senescence-associated β-galactosidase (SA β-Gal) staining

The staining was performed essentially as described by Dimri *et al*. [50]. Cells were seeded at 20% density as triplets. The next day cells were treated with the indicated compounds for different incubation times. Afterwards, the cells were washed with PBS and fixed for 5 min in 1% glutardialdehyde. Fixed cells were washed with PBS and incubated with fresh SA β-Gal staining solution [40 mM citric acid/sodium phosphate buffer (pH 6.0) containing 1 mg X-gal (5-bromo-4-chloro-3-indolyl-b-D-galactopyranoside)/ml, 5 mM potassium ferrocyanide 5 mM potassium ferricyanide, 150 mM NaCl, and 2 mM MgCl_2_] at 37°C without CO_2_. The staining solution contains X-Gal, a galactopyranosid, which is converted by an active galactosidase into a blue colorant. After overnight incubation blue stained cells were detected and counted by light microscopy [50, 51]. 200 cells per well were counted and the average of triplets is diagramed. Used compounds are: 1 nM (supraphysiological androgen level = SAL) and 1 pM R1881 (low androgen level = LAL) (Methyltrionolone, Perkin Elmer); 10 nM DHT (Sigma); 1 μM Src-Inhibitor PP2 (4-amino-5-(4-chloophenyl)-7-(t-butyl)Pyrazolo(3,4-d)pyrimidine, Calbiochem); 1 μM Akt-Inhibitor (1 L6-Hydroxymethyl-chiro-Insoitol-2-(R)-2-O-methyl-3-O-octadecyl-sn-glycerocarbonat, Calbiochem) and 1 nM rapamycin as an inhibitor of mTOR (LC Laboratories).

### Detection of senescence-associated heterochromatic foci (SAHF)

Cells were seeded as duplicates in RPMI at 10% confluence. After 24 h the cells were treated with different compounds for 3 d followed by washing with PBS and collected via trypsinization. DNA was visualized by DAPI (4′,6-Diamidino-2-phenylindol) at 1 μg/ml after dropping cells on glass slides and dehydration. Images were taken by fluorescence microscopy.

### FACS analysis

LNCaP cells were seeded as duplicates in RPMI at 10% confluence. After 24 h cells were treated with different compounds for 3 d. For FACS analysis cells were trypsinized, washed twice with PBS and fixed for 3 h with 70% ethanol at -20°C. Afterwards cells were incubated for 45 min at 4°C while rotating with staining solution (2.5 μg/ml propidium iodide, 0.1 mg/ml RNAse A and 0.05% Triton X-100). 10 000 cells were analyzed with CyFlow ML cytometer (Partec, Muenster, Germany) and the cell cycle phases were determined by Cylchred. The percentage of cells in the different cell cycle phases is indicated.

### MTT viability assay

LNCaP cells were seeded as triplets in RPMI at 5% confluence on 96-well plates. The next day cells were treated with the different compounds for 3 d followed by the addition of MTT-solution (cell growth determination Kit MTT based Marker, Sigma) and 4 h incubation at 37°C. Afterwards, the media/MTT-solution was removed, MTT-solvent was added and cells were measured at a wavelength of 570 nM (reference wavelength: 690 nM) via an ELISA-Reader.

### Western blot analysis

After 3 d of treatment proteins extracts were obtained via NETN buffer (200 mM NaCl, 20 mM Tris/HCl pH = 8.0, 1 mM EDTA, 0.5% NP40) and freezing-thawing using liquid nitrogen. Proteins were loaded on SDS-PAGEs with different acrylamid content, specific for the size of the investigated proteins. Visualization of proteins blotted on the membrane were performed by different primary antibodies [α-Tubulin, β-Actin, pRb (Abcam); p21, p53, ac p53 (Lys 379), p16, Cyclin D1, phospho-pRb (Ser 807/811), Src, phospho-Src (Tyr 416), Akt, phospho-Akt (Ser 473; Cell Signaling); E2F1 (Santa Cruz); LC3 (Sigma)] and secondary antibodies [anti-mouse, anti-rabbit (Santa Cruz)] coupled with horseradish peroxidase with enhanced chemiluminescence. Quantification of the bands was performed by the Labimage D1 program.

### Quantitative reverse transcription PCR (qRT-PCR)

RNA was isolated using peqGOLD TriFast (Peqlab) according to the manufacturer’s protocol. RNA was used in a one-step qRT-PCR reaction using the SuperScript III Platinum SYBR Green One-Step qRT-PCR Kit (Invitrogen) with the following primer sequences (indicated as 5′ → 3′):

p14 forw. CCTGGAGGCGGCGAGAAC, rev. CAGCACGAGGGCCACAGC;

p16 forw. CTTGCCTGGAAAGATACCG, rev. CCCTCCTCTTTCTTCCTCC;

p21 forw. TCGACTTTGTCACCGAGACACCAC, rev. CAGGTCCACATGGTCTTCCTCTG;

Atg3 forw. GCCCCAGGATGCAGAATGTG, rev. CAATTCTTCCCCTGTAGCCCATTG;

Atg5 forw. GCTTCGAGATGTGTGGTTTGGACG, rev. CCAAGGAAGAGCTGAACTTGATGC,

Atg7 forw. CAGTTTGCCCCTTTTAGTCAGTGCC, rev. AGCTTCATCCAGCCGATACTCGTTC,

β-actin forw. ACAGAGCCTCGCCTTTGCCGA, rev. CACGATGGAGGGGAAGACG;

Beclin 1 forw. CAGGTGAGCTTCGTGTGCC, rev. CCTGGCTGGGGGGATGAATC;

CyclinD1 forw. TCAACCTAAGTTCGGTTCCGATG, rev. GTCAGCCTCCACACTCTTGC;

E2F1: Fw: GCAGAGCAGATGGTTATGG, rev. GATCTGAAAGTTCTCCGAAGAG:

GAPDH forw. GTGAACCATGAGAAGTATGACAAC, rev. GAGTCCTTCCACGATACC;

ID1 forw. GGTAAACGTGCTGCTCTACGACATG, rev. CTCCAGCACGTCATCGACTACATC;

LC3A forw. CGAGTTGGTCAAGATCATCCGGC, rev. GCTCGTAGATGTCCGCGATGGGCG,

LC3B forw. TAGAGCGATACAAGGGGGAGAAGC, rev. TGTGTCGTTCACCAACAGGAAG and

PI3K forw. GCTTGGAAGGGAAGAGAGAACAAAAGAG, rev. CTTGGGCATTCCTGGGCAG.

Obtained data analyses were normalized to β-actin or GAPDH mRNA levels and diagramed as fold induction. Results were analyzed via the ∆∆Ct-method.

### siRNA transfection

To analyze the expression of specific genes in eukaryotic cells, stealth RNAi (Invitrogen) was used. LNCaP cells were trypsinized and with 10–20 nM siRNA transiently transfected by electroporation. As negative control srcambled siRNA, and additionally one sample without any siRNA were used. The knockdown efficiency was detected via qRT-PCR to analyze the specific gene expression. The transfected cells were treated with different compounds 24 h after electroporation and the induction of cellular senescence was analyzed.

### *Ex vivo*human prostate tissue analysis

Prostate cancer tissue derived from prostatectomy was pathological examined and as 5 x 5 mm pieces cultured with the different hormones in RPMI (1640) with 10% FCS, 1% penicillin/streptomycin, 1% sodium pyruvate and 25 mM HEPES (pH 7.8) for 2 d. Afterwards, the PCa tissue was cut in 5-10 μM slices via a cryotome and SA β-gal staining and RNA isolation followed as described before. Ethical approval was granted (3286-11/11). Gleason scores were between 7 and 9. Pre-surgery PSA values were between 4 and 16 ng/ml.

### 3D - interphase fluorescence in situ hybridization (iFISH)

3D-iFISH in LNCaP cell nuclei of untreated or treated cell with SAL for three days was performed as described earlier for lymphocytes [52]. DNA probes for the region q11.1-qter that include the E2F1 gene locus were obtained from the Multicolor Chromosome Banding (MCB) probes library (particularly, MCB 20–3 DNA probe was used). Overlap of chromosome 20 and SAHFs was found in 57 from 88 totally analyzed cells. DAPI staining was used to detect SAHFs. Overlap of FISH-signals and SAHFs was evaluated using the Cell^P software (Olympus).

## Electronic supplementary material

Additional file 1: Figure S1: Detection of the SA‒beta Gal activity comparing three and six days of incubation with low (LAL) or supraphysiological (SAL) androgen levels in LNCaP cells. Similar experimental setup as in Figure 1A. The level of senescent cells is not increased with longer treatment times. (DOC 42 KB)

Additional file 2: Figure S2: Detection ofthe SA‒beta Gal activity comparing three and six days of incubation with low (LAL) or supraphysiological (SAL) androgen levels in PC3‒AR cells or PC3-tet‒AR cells kindly provided by Dr. Volpert (Mirochnik et al. [47]). Similar experimental setup as in Figure 1A. Androgens mediate the induction of cellular senescence. A) Level of senescent cells after 3 or 6 days of treatment. B) Doxycyclin inducible expression of the human AR and AR‒dependent as well androgen‒dependent induction of cellular senescence. (DOC 90 KB)

Additional file 3: Figure S3: Changes of the indicated factors by androgen treatment for 6 days using LNCaP cells were analyzed by (A) qRT‒PCR and (B) by Western blotting similarly as described in Figure 4. β-actin was used as loading control. Quantification of the bands was realized via Labimage D1 and the expression levels of the target proteins were normalized and given as band intensity to the loading control β-actin, untreated sample was set arbitrarily as one. C: solvent control (DMSO). (DOC 98 KB)

Additional file 4: Figure S4: Detection of p21 and E2F1 mRNA and protein levels in PC3‒AR cells after in response LAL or SAL androgen levels detected by (A) qRT-PCR or (B) Western blotting, respectively. The p21 mRNA levels are increased after SAL whereas no significant changes of E2F1 were observed after androgen treatment for 72 hours. (DOC 66 KB)

Additional file 5: Figure S5: Detection of MEK1/2 phosphorylation in response LAL or SAL androgen levels in LNCaP cells detected by Western blotting. No significant changes of phosphorylation level of ERK1/2 were observed after androgen treatment for 72 hours. C: solvent control (DMSO). (DOC 59 KB)

## Abbreviations

AR: Androgen receptor
CRPCa: Castration resistant PCa
DHT: Dihydrotestosterone
DMSO: Dimethyl sulfoxide
iFISH: Interphase fluorescence *in situ* hybridyzation
LAL: Low androgen levels
mTOR: Mammalian target of rapamycin
PCa: Prostate cancer
PSA: Prostate specific antigen
R1881: Methyltrienolone
SA β-Gal: Senescence-associated beta-galactosidase
SAHF: Senescence-associated heterochromatic foci
SAL: Supraphysiological androgen levels
wt: Wild-type.

## References

[1] Jemal A, Siegel R, Xu J, Ward E (2010). Cancer statistics, 2010. CA Cancer J Clin.

[2] Cronauer MV, Culig Z (2012). Molecular aspects of prostate cancer. World J Urol.

[3] Dai WS, Kuller LH, LaPorte RE, Gutai JP, Falvo-Gerard L, Caggiula A (1981). The epidemiology of plasma testosterone levels in middle aged men. Am J Epidemiol.

[4] Prehn RT (1999). On the prevention and therapy of prostate cancer by androgen administration. Cancer Res.

[5] Morley JE, Kaiser FE, Perry HM, Patrick P, Morley PM, Stauber PM, Vellas B, Baumgartner RN, Garry PJ (1997). Longitudinal changes in testosterone, luteinizing hormone, and follicle-stimulating hormone in healthy older men. Metabolism.

[6] Umekita Y, Hiipakka RA, Kokontis JM, Liao S (1996). Human prostate tumor growth in athymic mice: inhibition by androgens and stimulation by finasteride. Proc Natl Acad Sci U S A.

[7] Calabrese EJ (2001). Androgens: biphasic dose responses. Crit Rev Toxicol.

[8] Denmeade SR, Isaacs JT (2010). Bipolar androgen therapy: the rationale for rapid cycling of supraphysiologic androgen/ablation in men with castration resistant prostate cancer. Prostate.

[9] Niu Y, Altuwaijri S, Lai KP, Wu CT, Ricke WA, Messing EM, Yao J, Yeh S, Cang C (2008). Androgen receptor is a tumor suppressor and proliferator in prostate cancer. Proc Natl Acad Sci U S A.

[10] Campisi J (2001). Cellular senescence as a tumor-suppressor mechanism. Trends Cell Biol.

[11] Shay JW, Roninson IB (2004). Hallmarks of senescence in carcinogenesis and cancer therapy. Oncogene.

[12] Storer M, Mas A, Robert-Moreno A, Pecoraro M, Ortells MC, Di Giacomo V, Yosef R, Pilpel N, Krizhanovsky V, Sharpe J, Keyes WM (2013). Senescence is a developmental mechanism that contributes to embryonic growth and patterning. Cell.

[13] Muñoz-Espín D, Cañamero M, Maraver A, Gómez-López G, Contreras J, Murillo-Cuesta S, Rodríguez-Baeza A, Varela-Nieto I, Ruberte J, Collado M, Serrano M (2013). Programmed cell senescence during mammalian embryonic development. Cell.

[14] Collado M, Gil J, Efeyan A, Guerra C, Schuhmacher AJ, Barrada M, Benguria A, Zaballos A, Flores JM, Barbacid M, Beach D, Serrano M (2005). Senescence in premalignant tumours. Nature.

[15] Schmitt CA (2007). Cellular senescence and cancer treatment. Biochim Biophys Acta.

[16] Wei W, Hemmer RM, Sedivy JM (2001). Role of p14(ARF) in replicative and induced senescence in human fibroblasts. Mol Cell Biol.

[17] Krtolica A, Campisi J (2002). Cancer and aging: a model for the cancer promoting effects of the aging stroma. Int J Biochem Cell Biol.

[18] Schwarze SR, Fu VX, Desotelle JA, Kenowsky ML, Jarrard DF (2005). The identification of senescence-specific genes during the induction of senescence in prostate cancer cells. Neoplasia.

[19] Campisi J, d’Adda di Fagagna F (2007). Cellular senescence: when bad things happen to good cells. Nat Rev Mol Cell Biol.

[20] Gamerdinger M, Hajieva P, Kaya AM, Wolfrum U, Hartl FU, Behl C (2009). Protein quality control during aging involves recruitment of the macroautophagy pathway by BAG3. EMBO J.

[21] Young ARJ, Narita M, Ferreira M, Kirschner K, Sadaie M, Darot JFJ, Arakawa S, Shimizu S, Watt FM, Narita M (2009). Autophagy mediates the mitotic senescence transition. Genes Dev.

[22] Narita M (2010). Quality and quantity control of proteins in senescence. Aging.

[23] Peterziel H, Mirk S, Schonert A, Becker M, Klocker H, Cato ACB (1999). Rapid signaling by androgen receptor in prostate cancer cells. Oncogene.

[24] Kang Z, Jänne OA, Palvimo JJ (2004). Coregulator recruitment and histone modifications in transcriptional regulation by the androgen receptor. Mol Endocrinol.

[25] Hammes SR, Levin ER (2007). Extra-nuclear steroid receptors: nature and function. Endocr Rev.

[26] Migliaccio A, Castoria G, Di Domenico M, de Falco A, Lombardi M, Barone MV, Ametrano D, Zannini MS, Abbondanza C, Auricchio F (2000). Steroid-induced androgen receptor-estradiol receptor beta-Src complex triggers prostate cancer cell proliferation. EMBO J.

[27] Migliaccio A, Di Domenico M, Castoria G, Nanayakkara M, Lombardi M, de Falco A, Bilancio A, Varricchio L, Ciociola A, Auricchio F (2005). Steroid receptor regulation of epidermal growth factor signaling through Src in breast and prostate cancer cells: steroid antagonist action. Cancer Res.

[28] Jenster G (1999). The role of the androgen receptor in the development and progression of prostate cancer. Semin Oncol.

[29] Sonnenschein C, Olea N, Pasanen ME, Soto AM (1989). Negative controls of cell proliferation: human prostate cancer cells and androgens. Cancer Res.

[30] Bonne C, Raynaud J-P (1976). Assay of androgen binding sites by exchange with methyltrienolone (R1881). Steroids.

[31] Schayowitz A, Sabnis G, Goloubeva O, Njar VC, Brodie AM (2010). Prolonging hormone sensitivity in prostate cancer xenografts through dual inhibition of AR and mTOR. Br J Cancer.

[32] Kaufman JM, Vermeulen A (2005). The decline of androgen levels in elderly men and its clinical and therapeutic implications. Endocr Rev.

[33] Sakaguchi K, Herrera JE, Saito S, Miki T, Bustin M, Vassilev A, Anderson CW, Appella E (1998). DNA damage activates p53 through a phosphorylation-acetylation cascade. Genes Dev.

[34] Narita M, Nune S, Heard E, Narita M, Lin AW, Hearn SA, Spector DL, Hannon GJ, Lowe SW (2003). Rb-mediated heterochromatin formation and silencing of E2F target genes during cellular senescence. Cell.

[35] Shaw RJ (2009). LKB1 and AMP-activated kinase control of mTOR signaling and growth. Acta Physiol.

[36] Sabatini DM (2006). mTOR and cancer: insights into a complex relationship. Nat Rev Cancer.

[37] Kroemer G, Marino G, Levine B (2010). Autophagy and the integrated stress response. Mol Cell.

[38] Gottlieb RA, Mentzer RM (2010). Autophagy during cardiac stress: joys and frustrations of autophagy. Annu Rev Physiol.

[39] Isaacs JT, D’Antonio JM, Chen S, Antony L, Dalrymple SP, Ndikuyeze GH, Luo J, Denmeade SR (2012). Adaptive auto-regulation of androgen receptor provides a paradigm shifting rationale for bipolar androgen therapy (BAT) for castrate resistant human prostate cancer. Prostate.

[40] Raynaud JP (2006). Prostate cancer risk in testosterone-treated men. J Steroid Biochem Mol Biol.

[41] Stattin P, Lumme S, Tenkanen L, Alfthan H, Jellum E, Hallmans G, Thorensen S, Hakulinen T, Luostarinen T, Lehtinen M, Dillner J, Stenman UH, Stenman M, Hakama M (2004). High levels of circulating testosterone are not associated with increased prostate cancer risk; a pooled prospective study. Int J Cancer.

[42] Chuu C-P, Kokontis JM, Hiipakka RA, Fukuchi J, Lin H-P, Lin C-Y, Huo C, Su L-C, Liao S (2011). Androgen suppresses proliferation of castration-resistant LNCaP 104-R2 prostate cancer cells through androgen receptor, Skp2, and c-Myc. Cancer Sci.

[43] de Launoit Y, Veilleux R, Dufour M, Simard J, Labrie F (1991). Characteristics of the biphasic action of androgens and of the potent antiproliferative effects of the new pure antiestrogen EM-139 on cell cycle kinetic parameters in LNCaP human prostatic cancer cells. Cancer Res.

[44] Dehm SM, Tindall DJ (2007). Androgen receptor structural and functional elements: role and regulation in prostate cancer. Mol Endocrinol.

[45] Johnson DG, Ohatani K, Nevis JR (1994). Autoregulatory control of E2F1 expression in response to positive and negative regulators of cell cycle progression. Genes Dev.

[46] Fousteris MA, Schubert U, Roell D, Roediger J, Ballis N, Nikolaropoulos SS, Baniahmad A, Giannis A (2010). 20-Aminosteroids as a novel class of selective and complete androgen receptor antagonists and inhibitors of prostate cancer cell growth. Bioorg Med Chem.

[47] Mirochnik Y, Veliceasa D, Williams L, Maxwell K, Yemelyanov A, Budunova I, Volpert OV (2012). Androgen receptor drives cellular senescence. PLoS One.

[48] Jarrard DF, Sarkar S, Shi Y, Yeager TR, Magrane G, Kinoshita H, Nassif N, Meisner L, Newton MA, Waldman FM, Reznikoff CA (1999). p16/pRb pathway alterations are required for bypassing senescence in human prostate epithelial cells. Cancer Res.

[49] Papaioannou M, Schleich S, Prade I, Degen S, Roell D, Schubert U, Tanner T, Claessens F, Matusch R, Baniahmad A (2009). The natural compound atraric acid is an antagonist of the human androgen receptor inhibiting cellular invasiveness and prostate cancer cell growth. J Cell Mol Med.

[50] Dimri GP, Lee X, Basile G, Acosta M, Scott G, Roskelley C, Medrano EE, Linskens M, Rubelj I, Pereira-Smith O, Peacocket M, Campisi J (1995). A biomarker that identifies senescent human cells in culture and in aging skin in vivo. Proc Natl Acad Sci U S A.

[51] Lorenz V, Hessenkemper W, Rödiger J, Kyrylenko S, Kraft F, Baniahmad A (2011). Sodium butyrate induces cellular senescence in neuroblastoma and prostate cancer cells. Hormone Mol Biol Clin Invest.

[52] Manvelyan M, Hunstig F, Mrasek K, Bhatt S, Pellestor F, Weise A, Liehr T (2008). Position of chromosomes 18, 19, 21 and 22 in 3D-preserved interphase nuclei of human and gorilla and white hand gibbon. Mol Cytogenet.

