# Supplementary figures and images for: Supraphysiological androgen levels induce cellular senescence in human prostate cancer cells through the Src-Akt pathway
Source: Mol Cancer. 2014 Sep 12;13:214. doi: 10.1186/1476-4598-13-214 (PMC4171558; doi:10.1186/1476-4598-13-214)

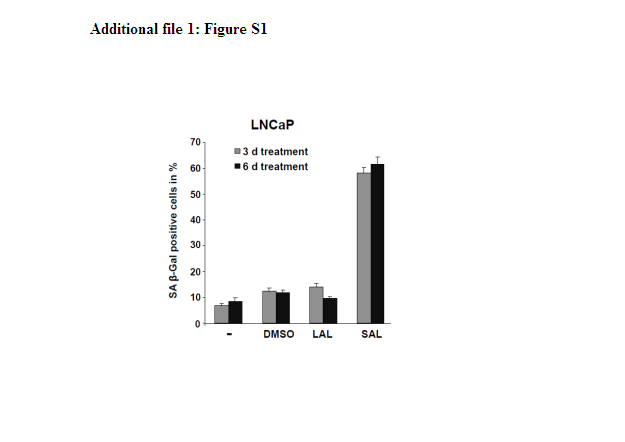

Supplement: Supplementary file 1 — Additional file 1: Figure S1: Detection of the SA‒beta Gal activity comparing three and six days of incubation with low (LAL) or supraphysiological (SAL) androgen levels in LNCaP cells. Similar experimental setup as in Figure 1A. The level of senescent cells is not increased with longer treatment times. (DOC 42 KB) [file 12943_2014_1413_MOESM1_ESM.doc]

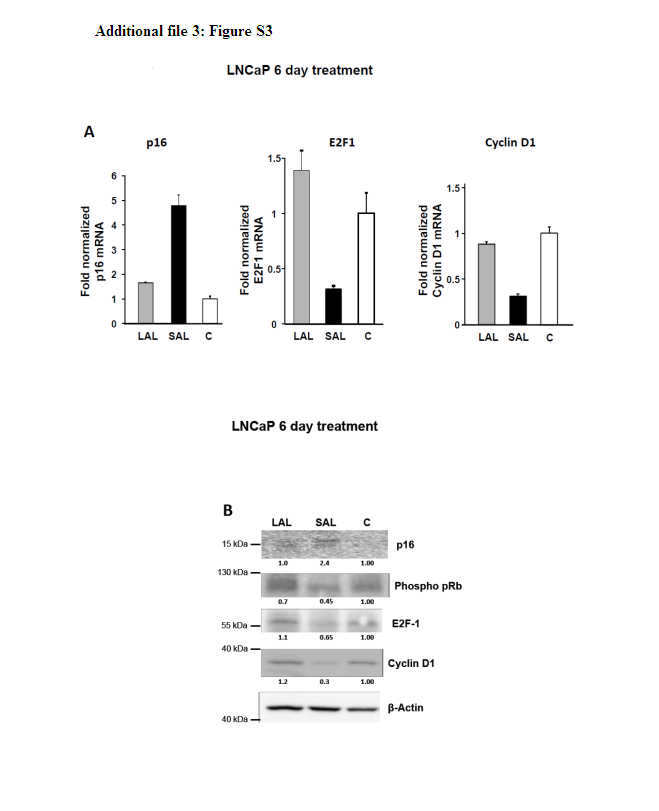

Supplement: Supplementary file 3 — Additional file 3: Figure S3: Changes of the indicated factors by androgen treatment for 6 days using LNCaP cells were analyzed by (A) qRT‒PCR and (B) by Western blotting similarly as described in Figure 4. β-actin was used as loading control. Quantification of the bands was realized via Labimage D1 and the expression levels of the target proteins were normalized and given as band intensity to the loading control β-actin, untreated sample was set arbitrarily as one. C: solvent control (DMSO). (DOC 98 KB) [file 12943_2014_1413_MOESM3_ESM.doc]

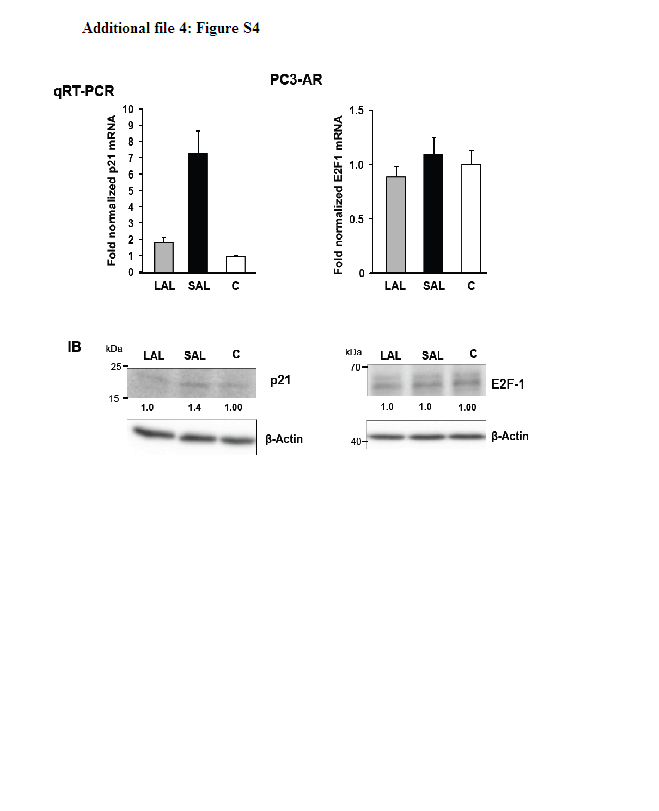

Supplement: Supplementary file 4 — Additional file 4: Figure S4: Detection of p21 and E2F1 mRNA and protein levels in PC3‒AR cells after in response LAL or SAL androgen levels detected by (A) qRT-PCR or (B) Western blotting, respectively. The p21 mRNA levels are increased after SAL whereas no significant changes of E2F1 were observed after androgen treatment for 72 hours. (DOC 66 KB) [file 12943_2014_1413_MOESM4_ESM.doc]

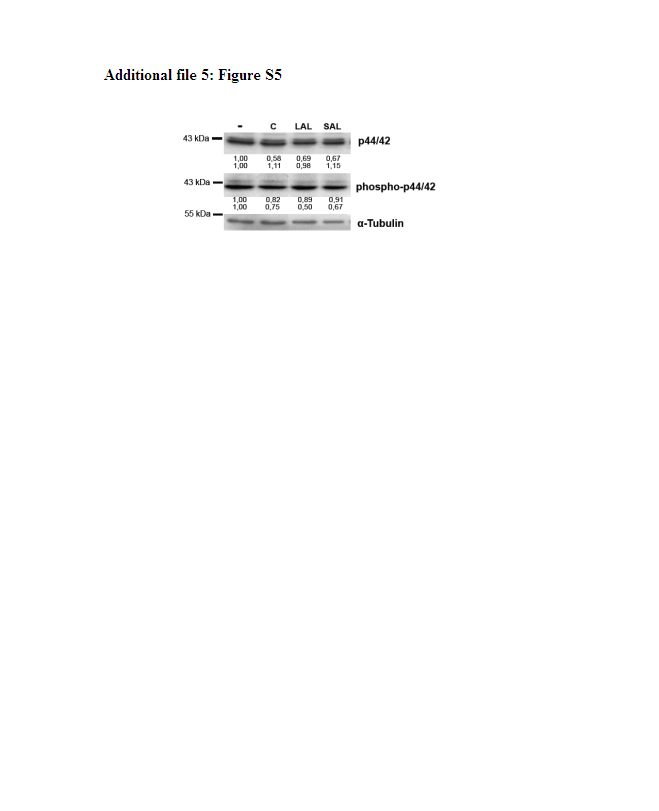

Supplement: Supplementary file 5 — Additional file 5: Figure S5: Detection of MEK1/2 phosphorylation in response LAL or SAL androgen levels in LNCaP cells detected by Western blotting. No significant changes of phosphorylation level of ERK1/2 were observed after androgen treatment for 72 hours. C: solvent control (DMSO). (DOC 59 KB) [file 12943_2014_1413_MOESM5_ESM.doc]
