# Supplementary material for: Supraphysiological androgen levels induce cellular senescence in human prostate cancer cells through the Src-Akt pathway
Source: Mol Cancer. 2014 Sep 12;13:214. doi: 10.1186/1476-4598-13-214 (PMC4171558; doi:10.1186/1476-4598-13-214)
Supplement: Supplementary file 2 — Additional file 2: Figure S2: Detection ofthe SA‒beta Gal activity comparing three and six days of incubation with low (LAL) or supraphysiological (SAL) androgen levels in PC3‒AR cells or PC3-tet‒AR cells kindly provided by Dr. Volpert (Mirochnik et al. [47]). Similar experimental setup as in Figure 1A. Androgens mediate the induction of cellular senescence. A) Level of senescent cells after 3 or 6 days of treatment. B) Doxycyclin inducible expression of the human AR and AR‒dependent as well androgen‒dependent induction of cellular senescence. (DOC 90 KB) [file 12943_2014_1413_MOESM2_ESM.doc]

Additional file 2: Figure S2
